# Exome sequencing study in patients with multiple sclerosis reveals variants associated with disease course

**DOI:** 10.1186/s12974-018-1307-1

**Published:** 2018-09-14

**Authors:** Elia Gil-Varea, Elena Urcelay, Carles Vilariño-Güell, Carme Costa, Luciana Midaglia, Fuencisla Matesanz, Alfredo Rodríguez-Antigüedad, Jorge Oksenberg, Laura Espino-Paisan, A. Dessa Sadovnick, Albert Saiz, Luisa M. Villar, Juan Antonio García-Merino, Lluís Ramió-Torrentà, Juan Carlos Triviño, Ester Quintana, René Robles, Antonio Sánchez-López, Rafael Arroyo, Jose C. Alvarez-Cermeño, Angela Vidal-Jordana, Sunny Malhotra, Nicolas Fissolo, Xavier Montalban, Manuel Comabella

**Affiliations:** 1 Servei de Neurologia-Neuroimmunologia, Centre d’Esclerosi Múltiple de Catalunya (Cemcat), Institut de Recerca Vall d’Hebron (VHIR), Hospital Universitari Vall d’Hebron, Universitat Autònoma de Barcelona, Barcelona, Spain; 2 Immunology Department, Hospital Clinico San Carlos, Instituto de Investigacion Sanitaria San Carlos (IdISSC), Madrid, Spain; 3 Department of Medical Genetics, University of British Columbia, Vancouver, Canada; 4 Department of Cell Biology and Immunology, Instituto de Parasitología y Biomedicina “López Neyra”, Consejo Superior de Investigaciones Científicas (IPBLN-CSIC), Granada, Spain; 5 Servicio de Neurología, Hospital Universitario Basurto-Osakidetza, Bilbao, Spain; 6 Department of Neurology, University of California, San Francisco, CA USA; 7 Neurology Service, Hospital Clinic and Institut d’Investigació Biomèdica Pi i Sunyer (IDIBAPS), Barcelona, Spain; 8 Departments of Immunology and Neurology, Multiple Sclerosis Unit, Hospital Ramon y Cajal, (IRYCIS), Madrid, Spain; 9 Neuroimmunology Unit, Puerta de Hierro University Hospital and Research Institute, Universidad Autónoma de Madrid, Madrid, Spain; 10 Neuroimmunology and Multiple Sclerosis Unit, Department of Neurology, Hospital Dr Josep Trueta, IDIBGI, University of Girona, Girona, Spain; 11 Genomic Systems, Valencia, Spain; 12 Servicio de Neurología, Hospital Universitario Quirón Salud, Madrid, Spain

**Keywords:** Multiple sclerosis, Immunology, Disease course, Exome sequencing, Polymorphisms, *CPXM2*, *IGSF9B*, *NLRP9*

## Abstract

**Background:**

It remains unclear whether disease course in multiple sclerosis (MS) is influenced by genetic polymorphisms. Here, we aimed to identify genetic variants associated with benign and aggressive disease courses in MS patients.

**Methods:**

MS patients were classified into benign and aggressive phenotypes according to clinical criteria. We performed exome sequencing in a discovery cohort, which included 20 MS patients, 10 with benign and 10 with aggressive disease course, and genotyping in 2 independent validation cohorts. The first validation cohort encompassed 194 MS patients, 107 with benign and 87 with aggressive phenotypes. The second validation cohort comprised 257 patients, of whom 224 patients had benign phenotypes and 33 aggressive disease courses. Brain immunohistochemistries were performed using disease course associated genes antibodies.

**Results:**

By means of single-nucleotide polymorphism (SNP) detection and comparison of allele frequencies between patients with benign and aggressive phenotypes, a total of 16 SNPs were selected for validation from the exome sequencing data in the discovery cohort. Meta-analysis of genotyping results in two validation cohorts revealed two polymorphisms, rs28469012 and rs10894768, significantly associated with disease course. SNP rs28469012 is located in *CPXM2* (carboxypeptidase X, M14 family, member 2) and was associated with aggressive disease course (uncorrected *p* value < 0.05). SNP rs10894768, which is positioned in *IGSF9B* (immunoglobulin superfamily member 9B) was associated with benign phenotype (uncorrected *p* value < 0.05). In addition, a trend for association with benign phenotype was observed for a third SNP, rs10423927, in *NLRP9* (NLR family pyrin domain containing 9). Brain immunohistochemistries in chronic active lesions from MS patients revealed expression of IGSF9B in astrocytes and macrophages/microglial cells, and expression of CPXM2 and NLRP9 restricted to brain macrophages/microglia.

**Conclusions:**

Genetic variants located in *CPXM2*, *IGSF9B*, and *NLRP9* have the potential to modulate disease course in MS patients and may be used as disease activity biomarkers to identify patients with divergent disease courses. Altogether, the reported results from this study support the influence of genetic factors in MS disease course and may help to better understand the complex molecular mechanisms underlying disease pathogenesis.

**Electronic supplementary material:**

The online version of this article (10.1186/s12974-018-1307-1) contains supplementary material, which is available to authorized users.

## Background

Multiple sclerosis (MS [MIM: 126200]) is a common disease of the central nervous system (CNS) of complex pathogenesis. Although the etiology of MS is unknown, it is assumed that both genetic and environmental factors, such as cigarette smoking, vitamin D deficiency, viral infections, or obesity, influence disease phenotype [1–3]. Additionally, there is increasing evidence that inherited epigenetic variation complements the role of the genetic predisposition in the susceptibility to develop MS [4]. Over the last years, genome-wide association studies (GWAS) have significantly contributed to the characterization of the MS genetic component, with the identification of more than 200 genetic variants outside the major histocompatibility complex (MHC) that influence the risk of developing MS [2–5]. Despite the growing knowledge of MS risk genes, still little is known about the disease-modifying genes that modulate MS course. Disease course in MS is extremely variable and patients may have relapse-onset or progressive clinical forms, and face benign or severe disease courses. Although the underlying cause of this disease variability remains elusive, evidence exists that it may be influenced by genetic factors [6, 7].

In the present study, we aimed to identify genes associated with MS disease course by first applying an exome sequencing approach to a discovery cohort of MS patients with benign and aggressive disease courses, followed by the validation of selected genetic variants in two independent cohorts of patients with divergent disease courses.

## Methods

### Discovery cohort

The discovery cohort comprised 20 MS patients classified according to their disease course into benign and aggressive phenotypes. Patients with benign phenotypes (*n* = 10) were defined as having an Expanded Disability Status Scale (EDSS) equal or lower than 3.0 after 15 or more years from disease onset [8] and never received MS therapies. Patients with aggressive disease courses (*n* = 10) reached an EDSS score equal or higher than 6.0 within the first 5 years after disease onset, regardless of treatment [9]. All patients included in the discovery cohort were recruited at the Centre d’Esclerosi Múltiple de Catalunya (Cemcat). Additional file 1: Table S1 summarizes demographic and main clinical characteristics of the discovery cohort.

### Exome sequencing

Genomic DNA was extracted from peripheral blood using standard methods. An exome sequencing approach was applied to the discovery cohort in order to identify genes associated with benign and aggressive disease courses. Exome sequencing was based on an Illumina HiSeq2000 sequencing platform and an Agilent’s SureSelect Target Enrichment System for 51 Mb. Sequencing was done with a 50× of coverage and reads were aligned against the human reference genome (GRCh37/hg19 assembly) using the Burrows-Wheeler Alignment tool (BWA) [10]. After reads mapping, low-quality reads and sequences flagged as PCR duplicates were removed from the BAM file using the Sequence Alignment/Map (SAM) [10] and Picard Tools. Unmasked variants were annotated considering all possible transcripts for each target gene, and in some cases variants located within a coding sequence when considering one isoform could be positioned within a non-coding region when considering another isoform, thus resulting also in the identification of intronic variants. Exome sequencing was performed in Sistemas Genómicos (Valencia, Spain).

### Selection of candidate single-nucleotide polymorphisms for validation

For the variant calling process, different algorithms were applied, including VarScan [11] and the *Genome Analysis Toolkit* (GATK) [12]. Python scripts were developed to combine variants. Variants annotation was based on Ensembl and NCBI databases. For the selection of significant variants, a Fisher exact test was applied to the benign and aggressive phenotypes. For prioritization and selection of the most promising variants, the following criteria were applied: (i) presence of two or more statistically significant variants per gene; (ii) odds ratio difference of the prevalence for the variant between disease phenotypes equal or higher than 2; (iii) absence of the variant in one disease phenotype and presence of the variant in ≥ 50% of patients belonging to the counterpart phenotype; (iv) missense variants, splice region variants, and variants reported as possible deleterious mutations; and (v) biological and functional relevance of the target genes to MS, as reported in the literature. A total of 16 independent variants satisfying 2 or more of the aforementioned criteria were selected for validation.

### Validation cohorts

Two independent cohorts with benign and aggressive disease courses were included in the study in order to validate the selected variants from the exome sequencing approach.

The first validation cohort included 194 MS patients from 7 MS centers [Bilbao (*n* = 56); UCSF (*n* = 55); Madrid—Hospital Clínico (*n* = 32); Barcelona—Hospital Clinic (*n* = 23); Madrid—Ramón y Cajal (*n* = 16); Madrid—Puerta de Hierro (*n* = 9); Girona (*n* = 3)]. Of these, 107 MS patients had benign phenotypes and 87 aggressive disease courses.

The second validation cohort consisted of 257 MS patients from Canada, 224 patients with benign phenotypes and 33 with aggressive disease courses. MS patients were ascertained through the Canadian Collaborative Project on the Genetic Susceptibility to Multiple Sclerosis (CCPGSMS) [13].

Clinical criteria to classify patients into benign and aggressive disease courses were the same as those applied to the discovery cohort, except for the second validation cohort in which treatment information on patients with benign disease course was not available. Similar to the discovery cohort, patients with benign phenotypes from the first validation cohort never received MS therapies. A summary of demographic and clinical characteristics of the first and second validation cohorts is shown in Additional file 1: Table S1.

The study was approved by the corresponding local ethics committees, and all participants provided informed consent.

### TaqMan OpenArray genotyping

Genotyping of selected variants in the first validation cohort was performed using an OpenArray technology (Thermo Fisher Scientific, Massachusetts, USA) and following the manufacturer’s instructions. Briefly, DNA samples were loaded into custom designed arrays using an OpenArray® AccuFill System (Thermo Fisher Scientific). QuantStudio™ 12K Flex system (Thermo Fisher Scientific) was used for sample amplification and fluorescent data collection. Hapmap samples with known genotype were included as internal controls of the process. Genotype was assigned using Taqman Genotyper Software (Thermo Fisher Scientific). Genotyping was performed by the Human Genotyping laboratory of the Spanish National Cancer Research Centre (CNIO).

### Sequenom MassARRAY genotyping

In the second validation cohort, selected variants were genotyped using a MassArray iPLEX platform (Sequenom, San Diego, CA, USA) as previously described [14].

### CPXM2, IGSF9B, and NLRP9 expression analysis in peripheral blood cells

Gene expression levels for *CPMX2*, *NLRP9*, and *IGSF9B* were determined by real-time PCR in peripheral blood mononuclear cells (PBMC) available from a subgroup of untreated MS patients from the first validation cohort. In order to avoid a confounding effect of disease course in the expression levels for these genes, analysis was restricted to the group of patients with aggressive disease course (*n* = 8 for *CPXM2*; *n* = 9 for *NLRP9*; *n* = 7 for *IGSF9B*). Briefly, PBMC were isolated by Ficoll-Isopaque density gradient centrifugation (Gibco BRL, Life Technologies LTD, Paisley, UK) and stored in liquid nitrogen until used. Total RNA was extracted from PBMC using TRIzol® reagent (Invitrogen, Carlsbad, CA) and cDNA synthesized using the High Capacity cDNA Archive kit (Applied Biosystems, Foster City, CA, USA). Messenger RNA expression levels for *CPMX2*, *NLRP9*, and *IGSF9B* were determined by real-time PCR using TaqMan® probes specific for each gene (Applied Biosystems, Foster City, CA, USA). The housekeeping gene glyceraldehyde-3-phosphate dehydrogenase (*GAPDH*) was used as an endogenous control (Applied Biosystems). Assays were run on the ABI PRISM® 7900HT system (Applied Biosystems) and data were analyzed using the 2^−∆∆CT^ method [15]. Results were expressed as fold change in gene expression in MS patients carrying the risk allele relative to non-carrier patients.

### Immunohistochemistry for CPXM2, IGSF9B, and NLRP9 in MS brain tissue

Paraffin-embedded brain samples of chronic active lesions from four MS patients were provided by the UK Multiple Sclerosis Tissue Bank and stained with hematoxylin and eosin (HE) and Klüver-Barrera (KB) for inflammation and demyelination assessment. Four-micrometer-thick, paraffin-embedded serial sections were deparaffined in xylene and rehydrated in alcohol. Endogenous peroxidase activity was blocked with hydrogen peroxide (2%), methanol (70%), and PBS for 20 min. Antigen retrieval was performed in TE buffer (1 M TrismaBase and 1 mM EDTA) (pH = 9) in the microwave. Non-specific protein binding was blocked with 0.2% of bovine albumin (BSA) in PBS. Sections were incubated overnight at 4 °C with the following primary antibodies: rabbit anti-CPXM2 (Biorbyt), rabbit anti-NLRP9 (Abcam), and rabbit anti-IGSG9B (Abcam). Samples were incubated for 1 h at room temperature with goat-anti rabbit HRP secondary antibody (Dakocytomation) and stainings were visualized with 3,3′diaminobenzidine (Sigma, St Louis, MO, USA) as a chromogenic substrate.

## Results

### Exome sequencing in the discovery cohort of patients with benign and aggressive disease courses and selection of candidate genetic variants

**Fig. 1:**
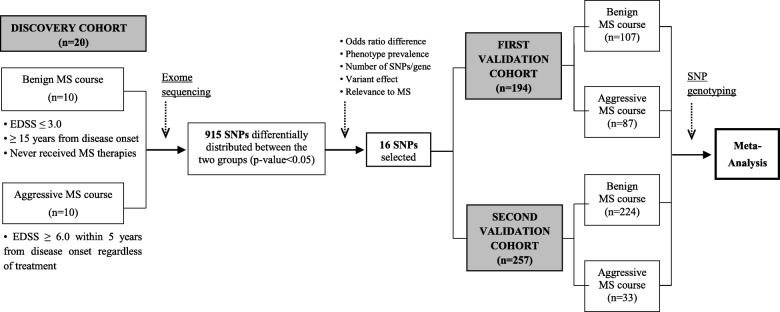
Flowchart showing the study design and analysis. Patients were classified according to their disease course into benign and aggressive MS, as described in the Methods. By means of exome sequencing, a total of 915 single-nucleotide polymorphisms (SNPs) were identified from 10 MS patients with benign and 10 with aggressive disease courses as being differentially distributed between both groups (discovery cohort). After applying several selection criteria on the list of 915 SNPs including odds ratio difference, phenotype prevalence, number of statistically significant SNPs per gene, type and variant effects on the predicted protein, and relevance of target genes to MS, a total of 16 SNPs were chosen for further validation in two independent cohorts of patients also classified into benign and aggressive phenotypes. The first validation cohort comprised 194 MS patients, 107 with benign and 87 with aggressive disease courses, and genotyping was conducting using an OpenArray technology. The second validation cohort consisted of 257 MS patients, 224 with benign and 33 with aggressive disease courses, and genotyping was performed on a MassArray iPLEX platform. Finally, a meta-analysis was performed in the two validation cohorts

A flowchart summarizing the main steps of the study design and analysis is depicted in Fig. 1. In order to identify genetic variants associated with disease course, we first performed exome sequencing in an initial cohort of 20 MS patients classified according to their disease course into benign (*n* = 10) and aggressive (*n* = 10). A total of 915 single-nucleotide polymorphisms (SNPs) were differentially distributed between MS patients with benign and aggressive disease courses (uncorrected *p* values < 0.05; data not shown). From the list of differentially distributed SNPs and after applying the selection criteria described in the “Methods” section, 16 SNPs were chosen for validation in two validation cohorts. Table 1 shows a description of the 16 selected SNPs from the discovery cohort, and Table 2 provides the results of the exome sequencing analysis for these 16 SNPs.

**Table 1:** Description and functional consequences of SNPs selected for validation from exome sequencing

SNP	Location (hg19)	Gene	Official full name	Variant effect
rs17844444 (G/A)	chr5:140,532,165	*PCDHB6*	Protocadherin Beta 6	Missense variant
rs17082236 (C/A)	chr6:152,470,752	*SYNE1*	Spectrin repeat containing nuclear envelope protein 1	Missense variant
rs10279499 (C/A)	chr7:92,733,766	*SAMD9*	Sterile alpha motif domain containing 9	Missense variant^a^
rs10488532 (C/T)	chr7:92,764,489	*SAMD9L*	Sterile alpha motif domain containing 9 like	Missense variant
rs2374639 (T/C)	chr7:92,985,252	*VPS50*	VPS50, EARP/GARPII complex subunit	Synonymous variant
rs17552167 (C/T)	chr7:148,529,922	*EZH2*	Enhancer of zeste 2 polycomb repressive complex 2 subunit	Intronic variant
rs28469012 (A/T)	chr10:125,622,263	*CPXM2*	Carboxypeptidase X, M14 family, member 2	Intronic variant
rs10894768 (C/G)	chr11:133,815,981	*IGSF9B*	Immunoglobulin superfamily member 9B	Synonymous variant
rs60252902 (G/A)	chr12:125,451,767	*DHX37*	DEAH-box helicase 37	Splice region variant^b^
rs3742130 (G/A)	chr13:99,907,341	*GPR18*	G protein-coupled receptor 18	Synonymous variant
rs9919887 (A/G)	chr14:95,911,008	*SYNE3*	Spectrin repeat containing nuclear envelope family member 3	Intronic variant
rs2230434 (C/T)	chr16:30,518,096	*ITGAL*	Integrin subunit alpha L	Synonymous variant
rs2070896 (T/C)	chr16:31,384,554	*ITGAX*	Integrin subunit alpha X	Intronic variant
rs35299026 (G/A)	chr19:49,318,380	*HSD17B14*	Hydroxysteroid 17-beta dehydrogenase 14	Missense variant^a^
rs10423927 (A/G)	chr19: 56,235,537	*NLRP9*	NLR family pyrin domain containing 9	Intronic variant
rs2254562 (T/C)	chr21:34,059,352	*SYNJ1*	Synaptojanin 1	Missense variant^a^

^a^Variants reported as possible deleterious mutations (based on SIFT and PolyPhen-2 algorithms)

^b^By definition, a splice region variant is a sequence variant in which a change has occurred within the region of the splice site, either within 1–3 bases of the exon or 3–8 bases of the intron [30]

**Table 2:** Summary of statistical analysis of selected SNPs in the discovery and validation cohorts

Selected variants			Discovery cohort (*n* = 20)			First validation cohort (*n* = 194)		Second validation cohort (*n* = 257)		Meta-analysis	
SNP	Gene	Minor allele	MAF, Benign (*n* = 10)	MAF, Aggressive (*n* = 10)	*p* value	MAF, Benign (*n* = 107)	MAF, Aggressive (*n* = 87)	MAF, Benign (*n* = 224)	MAF, Aggressive (*n* = 33)	Joint *p* value	OR (95% CI)
rs17844444	*PCDHB6*	A	0.10	0.40	0.03	0.15	0.15	0.18	0.14	0.52	
rs17082236	*SYNE1*	A	0.00	0.35	0.003	0.09	0.1	0.05	0.07	0.54	
rs10279499	*SAMD9*	A	0.45	0.00	0.0006	0.12	0.12	0.10	0.04	0.29	
rs10488532	*SAMD9L*	T	0.45	0.00	0.0006	0.12	0.14	0.12	0.06	0.79	
rs2374639	*VPS50*	C	0.60	0.06	0.003	0.27	0.31	0.27	0.15	(*)	
rs17552167	*EZH2*	T	0.30	0.00	0.007	0.07	0.06	0.08	0.12	0.63	
rs28469012	*CPXM2*	T	0.05	0.45	0.003	0.04	0.10	0.10	0.12	0.04	1.81 [1.03–3.18]
rs10894768	*IGSF9B*	G	0.50	0.05	0.001	0.35	0.28	0.36	0.27	0.04	0.70 [0.49–0.99]
rs60252902	*DHX37*	A	0.30	0.00	0.007	0.11	0.11	0.09	0.07	0.76	
rs3742130	*GPR18*	A	0.55	0.05	0.0005	0.26	0.27	0.23	0.19	0.80	
rs9919887	*SYNE3*	G	0.05	0.45	0.003	0.20	0.16	0.20	0.19	0.43	
rs2230434	*ITGAL*	T	0.00	0.25	0.01	0.11	0.09	0.09	0.07	0.32	
rs2070896	*ITGAX*	C	0.56	0.05	0.0006	0.37	0.38	0.33	0.32	0.85	
rs35299026	*HSD17B14*	A	0.25	0.00	0.01	0.05	0.07	0.05	0.06	0.31	
rs10423927	*NLRP9*	G	0.25	0.00	0.01	0.11	0.06	0.08	0.06	0.09	0.58 [0.31–1.09]
rs2254562	*SYNJ1*	C	0.05	0.45	0.003	0.30	0.29	0.29	0.35	0.65	

The minor allele frequencies (MAF) and uncorrected *p* values for each SNP selected from the discovery cohort are presented. For validation, heterogeneity tests and subsequent meta-analysis was performed in the two replication cohorts whenever possible. MAF and joint *p* values (calculated with Mantel-Haenszel test) are depicted for each SNP. In addition, odds ratio (OR) with their corresponding 95% confidence intervals (95% CI) are presented for SNPs with statistically significant results. *For SNP rs2374639, validation cohorts cannot be meta-analyzed due to high heterogeneity (*I*^2^ = 80%, *p* = 0.03). On a separate analysis, the first validation cohort shows replication of the effect observed in the discovery cohort, with a lower frequency of the minor allele in aggressive MS compared to benign MS [*p* = 0.04, OR = 0.47 (0.23–0.95)]. The second validation cohort shows an increased frequency of the minor allele in aggressive MS that is not statistically significant [*p* = 0.39, OR = 1.22 (0.77–1.94)]

### Validation of selected SNPs in two independent cohorts of patients with benign and aggressive disease courses

**Fig. 2:**
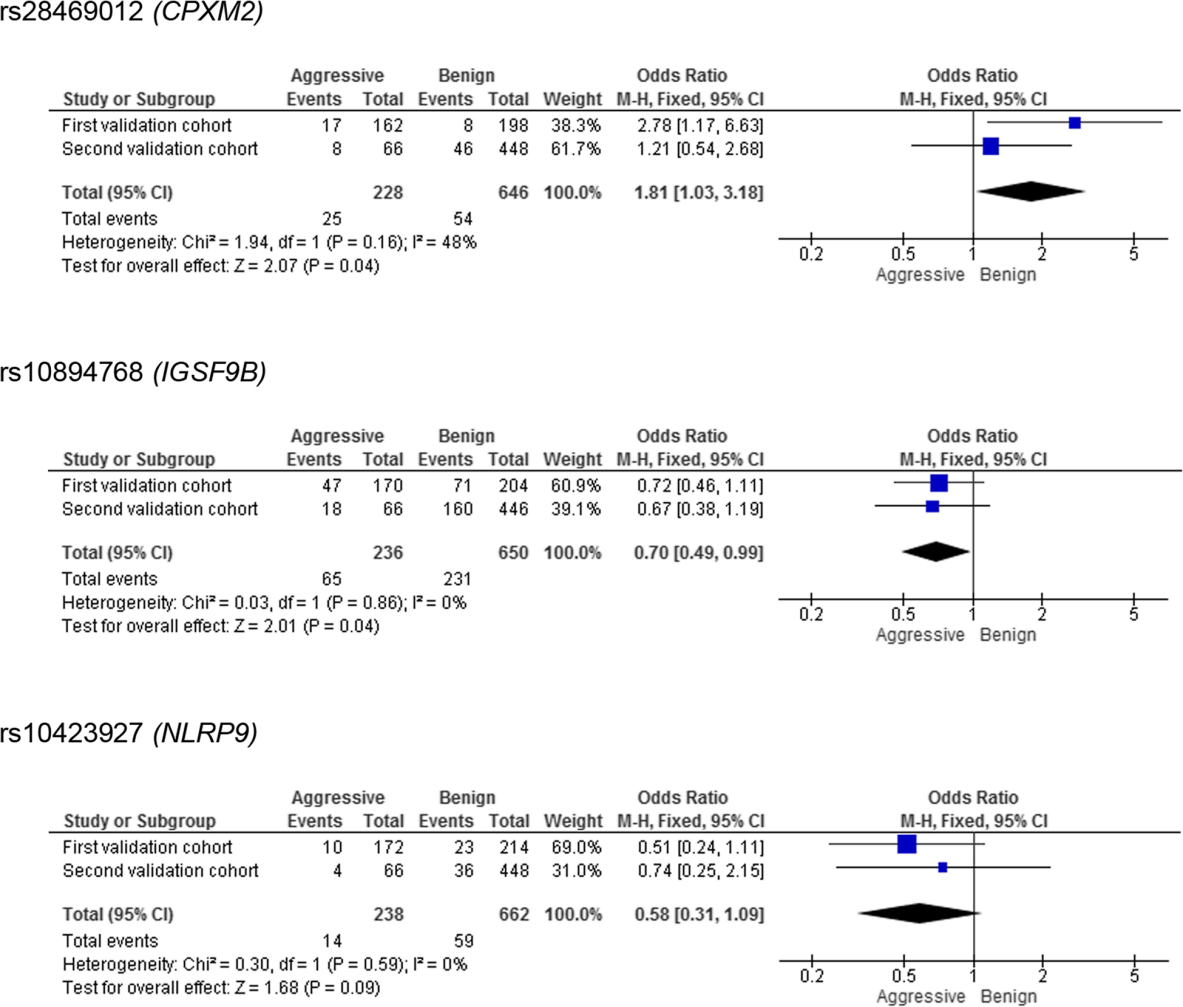
Meta-analysis for rs28469012 (*CPXM2*), rs10894768 (*IGSF9B*), and rs10423927 (*NLRP9*) in the two validation cohorts of patients with aggressive and benign disease courses. The figure depicts joint analyses for the first and second validation cohorts in each SNP, with homogeneity tests (*I*^2^) and tests for overall effects. The squares and horizontal lines correspond to the study specific odds ratios (ORs) and 95% confidence intervals (CI) respectively. The area of the squares reflects the study specific weight (inverse of the variance). The diamond represents the pooled ORs and 95% CI. *M*-*H* Mantel-Haenszel

Selected SNPs were genotyped in two independent validation cohorts composed of MS patients classified according to their disease course. A total of 194 MS patients were included in the first validation cohort and 257 MS patients in the second validation cohort. Meta-analysis of genotyping results in both cohorts identified two SNPs, rs28469012 and rs10894768, significantly associated with MS disease course. As shown in Fig. 2, the minor allele for rs28469012, an intronic SNP positioned in *CPXM2* (carboxypeptidase X, M14 family, member 2), was nominally significantly associated with more aggressive disease course [p-value; odds ratio (95% confidence interval): *p* = 0.04; 1.81 (1.03–3.18)] (Fig. 2). In this case, a modest degree of heterogeneity was found between both validation cohorts.

The minor allele for rs10894768, which corresponds to a synonymous SNP located in *IGSF9B* (immunoglobulin superfamily member 9B), was nominally significantly associated with benign disease course [*p* = 0.04; 0.70 (0.49–0.99)]. Meta-analysis of this variant supported the originally identified association with homogeneous outcomes for both cohorts (*I*^2^ = 0).

A trend for significance was observed in a third SNP, rs10423927, an intronic variant located in *NLRP9* (NLR family pyrin domain containing 9), with the minor allele associated with benign disease course [*p* = 0.09; 0.58 (0.31–1.09)]. Similar to the *IGSF9B* variant, meta-analysis for rs10423927 also supported the association in the discovery cohort and resulted in similar outcomes in both validation cohorts (*I*^2^ = 0).

It should be noted that rs2374639, a synonymous variant positioned in *VPS50* (VPS50, EARP/GARPII complex subunit), showed a significant association in the second validation cohort with the same protective effect observed in the discovery cohort [*p* = 0.04; 0.47 (0.23–0.95)]. However, the heterogeneity found between both validation cohorts did not allow to perform a joint analysis (Table 2; *I*^2^ = 80%).

Genotyping frequencies and results of statistical analysis in the two validation cohorts for all selected variants are provided in Table 2.

### Tissue-specific expression of IGSF9B, CPXM2, and NLRP9

In order to explore the functional consequences of the polymorphisms associated with MS disease course, we first investigated whether mRNA expression levels for *IGSF9B*, *CPXM2*, and *NLRP9* in PBMC differed between MS patients carrying the minor allele associated with disease course and non-carrier patients. *CPXM2* and *NLRP9* expression was not detected in PBMC from MS patients (data not shown). *IGSF9B* expression was detected in PBMC from MS patients, although no differences were observed between carriers and non-carriers of the minor allele associated with benign disease course (Additional file 1: Figure S1).

In order to determine whether the selected genetic variants associated with disease course could be modifying the expression of nearby genes in the region, we analyzed *cis*-expression quantitative trait loci (eQTLs) in 48 tissues from the Genotype-Tissue Expression (GTEx) project [16], lymphoblastoid cell lines in 465 individuals from the Geuvadis project [17], and trans-eQTLs from expression data of 9196 tumor samples in 33 cancer types from the PancanQTL study [18]. We observed correlation of the rs10894768 genotypes with *IGSF9B* expression in pancreatic tissue (*p* = 3.6 × 10^− 6^) from 171 individuals and thyroid tissue (*p* = 5.6 × 10^− 11^) from 323 individuals from the GTEx study [16]. The minor allele for this polymorphism, which was more represented in patients with benign disease course, was associated with lower *IGSF9B* expression (Additional file 1: Figure S2). Of note, *IGSF9B* was highly expressed in cerebellar hemisphere, cerebellum and also in cortex, hypothalamus, and spinal cord; however, no eQTLs were described for these tissues most likely due to the lower number of samples analyzed in brain tissue (105 samples from cerebellar hemisphere and 125 from cerebellum).

### IGSF9B, CPXM2, and NLRP9 are expressed in brain chronic active lesions from MS patients

**Fig. 3:**
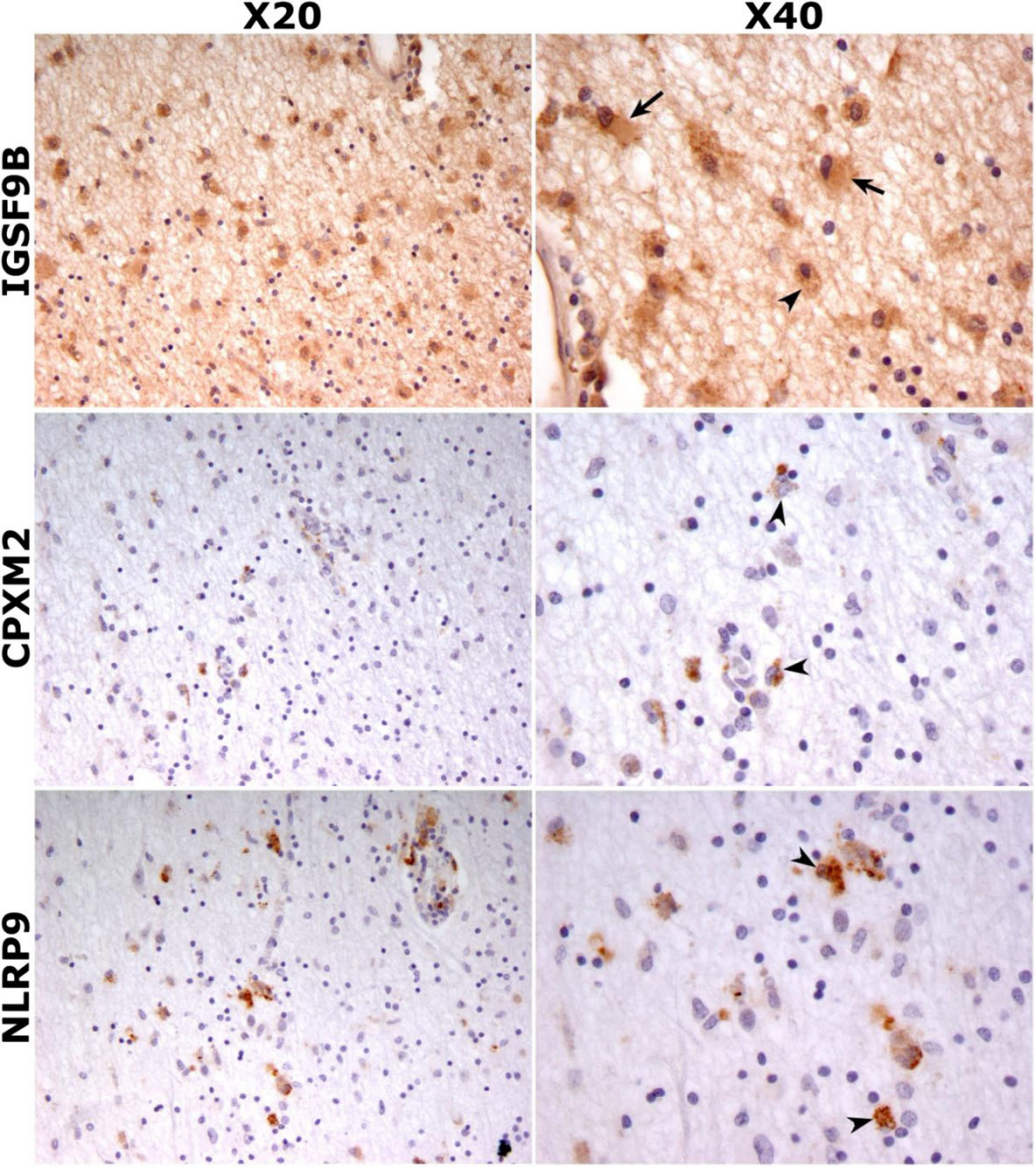
IGSF9B, CPXM2, and NLRP9 expression in MS brain tissue. Immunostainings for IGSF9B, CPXM2, and NLRP9 in the margins of MS brain chronic active lesions, where inflammatory activity is highest. IGSF9B expression was observed in astrocytes (arrows) and macrophages/microglia (arrow heads), whereas CPMX2 and NLRP9 immunostaining was only detected in macrophages/microglia (arrow heads). Photos were taken at × 20 and × 40

Based on the negative results observed for IGSF9B, CPXM2, and NLRP9 expression in peripheral blood, we wondered whether selected genes associated with MS disease course could play more specific roles at the CNS level by investigating their expression in brain tissue from patients. Figure 3 depicts the results of IGSF9B, CPXM2, and NLRP9 immunohistochemistries in brain tissue sections from chronic active lesions of MS patients. IGSF9B showed a diffuse neutrophil staining in both the gray and the white matter. The most intense IGSF9B staining was detected in the cytoplasm of microglia/macrophages and astrocytes located at the margins of MS lesions, where inflammatory activity is highest. IGSF9B immunostaining was also observed in less active areas of the lesions, albeit to a lesser degree compared to more active areas. CPXM2 and NLRP9 exhibited a similar and very discrete immunostaining. Their expression was restricted to the cytoplasm of few microglia/macrophages located at the margins of MS lesions showing the highest inflammatory activity (Fig. 3).

## Discussion

Exome sequencing has significantly contributed to the characterization of the genetic component of a number of common complex diseases [19]. In the present study, we aimed to identify genetic variants associated with MS disease course by applying, as a first step, an exome sequencing approach to a small discovery cohort of patients stratified according to benign and aggressive phenotypes. This initial approach led to the identification of a ranked list of candidate polymorphisms associated either with benign or aggressive MS disease courses. Despite the statistically significant associations of a large number of SNPs with MS disease course, in small discovery cohorts only strong statistically significant associations are likely to be real, and hence original findings should be better replicated in additional cohorts to eliminate the chances of false positive results. Based on these observations, in our study selected SNPs identified in the discovery cohort were further genotyped in two independent validation cohorts of patients classified according to similar criteria into benign and aggressive phenotypes.

Meta-analysis in the two validation cohorts revealed two polymorphisms, rs28469012 and rs10894768, as potential MS phenotype modifiers. The SNP rs28469012 is an intronic variant located in the *CPXM2* gene whose minor allele was associated with worse disease evolution. *CPXM2* codes for a member of the metallocarboxypeptidase family with potential roles in synaptic integrity [20]. Previous studies have associated the *CPXM2* gene to Alzheimer disease [21], Parkinson’s disease [20], and schizophrenia [22]. Although no studies of CPXM2 have been reported thus far in MS, it is interesting to mention that experimental autoimmune encephalomyelitis (EAE) mice deficient for another metallocarboxypeptidase that shares protein homology with CPXM2, carboxipeptidase N, had attenuated EAE disease course and reduced spinal cord inflammation and demyelination [23], data that indirectly support the association observed in our study between CPXM2 and aggressive MS phenotypes.

The synonymous exonic variant rs10894768 is positioned in the *IGSF9B* gene, and the minor allele for this polymorphism was more represented in MS patients with benign disease course. *IGSF9B* encodes a transmembrane immunoglobulin that has been reported to be highly expressed in GABAergic interneurons, where it may play a role promoting inhibitory synaptic development via the formation of a ternary complex with the postsynaptic scaffolding protein S-SCAM and the neuronal cell surface protein neuroligin 2 [24]. Similar to CPXM2, the role of IGSF9B in MS is unknown. However, considering that the GABAergic system is dysregulated in both MS and EAE and a selective loss of GABAergic interneurons has been reported in EAE [25], it is tempting to speculate that the finding of a higher frequency of genetic variants located in a gene that promotes maintenance of inhibitory synapses may result in more benign disease outcomes of MS patients. Noteworthy, MS brain tissue immunohistochemistry revealed IGSF9B expression in astrocytes, cells that are known to be involved in the formation and control of neuronal synapses [26].

Although rs28469012 and rs10894768 were the only polymorphisms whose association with MS disease course was validated, a trend for association with benign phenotypes was also observed for rs10423927, an intronic variant located in the *NLRP9* gene. Despite that little evidence exists in the literature regarding its function, NLRP9 belongs to the NOD-like receptor (NLR) family of inflammasomes, which are known to play critical roles both in innate and adaptive immunity and whose dysfunction has strongly been linked to autoimmune diseases [27]. Interestingly, a missense variant located in another member of the NLR family of inflammasomes, NLRP5, was recently found to be associated with higher disease severity scores, suggesting a role of NLR inflammasomes in MS disease course [28].

In an attempt to investigate the functional consequences of the genetic variants associated with benign and aggressive phenotypes, expression of IGSF9B, CPXM2, and NLRP9 was investigated at the gene and protein expression levels in PBMC and brain tissue respectively. Although not proven in the study, the negative results obtained in peripheral blood, with lack of expression of CPXM2 and NLRP9 in PBMC and no evidence of differences in *IGSF9B* expression between minor allele carriers and non-carriers for rs10894768, suggest that the genetic variants associated with disease course in MS may act by modulating the function of CNS cells such as macrophages/microglia and astrocytes, as supported by the immunohistochemistry studies in MS brain tissue showing expression for these genes in these particular cell types. Unfortunately, postmortem brain studies are not suitable for patient stratification to explore allele-specific gene expression differences. Furthermore, it could be possible that the MS course-associated allele of our reported SNPs confers increased ability to interact with certain environmental risk factors or impacts on chromatin structure by affecting epigenetic marks, including DNA methylation or histone modifications [29].

Finally, the finding that the minor allele of rs10894768, which is more represented in MS patients with benign outcomes, was associated with lower expression of *IGSF9B* in thyroid and pancreatic tissues supports the view that gene expression may be markedly different across tissues.

## Conclusions

In summary, we identified genetic variants in the *IGSF9B*, *CPXM2*, and *NLRP9* genes associated with benign and aggressive disease phenotypes in MS patients. Interestingly, the two genes that were validated in two independent cohorts of MS patients, *IGSF9B* and *CPXM2*, are known to play roles in CNS synapse integrity, findings that warrant additional studies to explore at the CNS level the potential functional consequences of the reported polymorphisms associated with MS disease course. Finally, aiming to provide a personalized medicine in MS, the reported polymorphisms may become disease activity biomarkers to identify MS patients with diverging disease courses.

## Additional file

Additional file 1: **Table S1.** Demographic and clinical characteristics of the MS patients with benign and aggressive disease courses. **Figure S1.** *IGSF9B* expression levels in PBMC from MS patients stratified according to the genetic variant associated with disease course. **Figure S2.** GTEx eQTLs of rs10894768 associations with *IGSF9B* expression in thyroid and pancreas tissues. (DOC 551 kb)

## Abbreviations

BWA: Burrows-Wheeler Alignment tool
CCPGSMS: Canadian Collaborative Project on the Genetic Susceptibility to Multiple Sclerosis
Cemcat: Centre d’Esclerosi Múltiple de Catalunya
CNIO: Spanish National Cancer Research Centre
CPXM2: Carboxypeptidase X, M14 family member 2
EAE: Experimental autoimmune encephalomyelitis
EDSS: Expanded Disability Status Scale
eQTL: *Cis*-expression quantitative trait loci
GATK: Genome analysis toolkit
GRCh37: Genome Reference Consortium Human Build 37
GWAS: Genome-wide association studies
IGSF9B: Immunoglobulin superfamily member 9B
KB: Klüver-Barrera
MS: Multiple sclerosis
NLR: NOD-like receptor
NLRP5: NLR family pyrin domain containing 5
NLRP9: NLR family pyrin domain containing 9
SAM: Sequence Alignment/Map
SNP: Single-nucleotide polymorphism
TE: Tris-ethylenediaminetetraacetic acid
UCSF: University of California, San Francisco
VPS50: VPS50, EARP/GARPII complex subunit

## References

[1] Ascherio A, Munger KL, Lünemann JD (2012). The initiation and prevention of multiple sclerosis. Nat Rev Neurol.

[2] Sawcer S, Hellenthal G, Pirinen M, Spencer CCA, Patsopoulos NA, Moutsianas L (2011). International multiple sclerosis genetics consortium (IMSGC) and Wellcome Trust case control consortium 2 (WTCCC2). Genetic risk and a primary role for cell-mediated immune mechanisms in multiple sclerosis. Nature.

[3] Beecham AH, Patsopoulos NA, Xifara DK, Davis MF, Kemppinen A, Cotsapas C (2013). International multiple sclerosis genetics consortium (IMSGC), Wellcome Trust case control consortium 2 (WTCCC2), international IBD genetics consortium (IIBDGC). Analysis of immune-related loci identifies 48 new susceptibility variants for multiple sclerosis. Nat Genet.

[4] Küçükali Cİ, Kürtüncü M, Çoban A, Çebi M, Tüzün E (2015). Epigenetics of multiple sclerosis: an updated review. Neuromolecular Med..

[5] Patsopoulos N, Baranzini SE, Santaniello A, Shoostari P, Cotsapas C, Wong G, et al. International Multiple Sclerosis Genetics Consortium (IMSGC). The Multiple Sclerosis Genomic Map: role of peripheral immune cells and resident microglia in susceptibility. Available at: https://www.biorxiv.org/content/early/2017/07/13/143933. Accessed 24 May 2018.

[6] Oksenberg JR, Barcellos LF (2005). Multiple sclerosis genetics: leaving no stone unturned. Genes Immun.

[7] Kantarci OH, de Andrade M, Weinshenker BG (2002). Identifying disease modifying genes in multiple sclerosis. J Neuroimmunol.

[8] Amato MP, Zipoli V, Goretti B, Portaccio E, De Caro MF, Ricchiuti L (2006). Benign multiple sclerosis: cognitive, psychological and social aspects in a clinical cohort. J Neurol.

[9] Menon S, Shirani A, Zhao Y, Oger J, Traboulsee A, Freedman MS (2013). Characterising aggressive multiple sclerosis. J Neurol Neurosurg Psychiatry.

[10] Li H, Durbin R (2009). Fast and accurate short read alignment with Burrows-Wheeler transform. Bioinformatics.

[11] Koboldt DC, Chen K, Wylie T, Larson DE, McLellan MD, Mardis ER (2009). VarScan: variant detection in massively parallel sequencing of individual and pooled samples. Bioinformatics.

[12] McKenna A, Hanna M, Banks E, Sivachenko A, Cibulskis K, Kernytsky A (2010). The genome analysis toolkit: a MapReduce framework for analyzing next-generation DNA sequencing data. Genome Res.

[13] Sadovnick AD, Risch NJ, Ebers GC (1998). Canadian collaborative project on genetic susceptibility to MS, phase 2: rationale and method. Can J Neurol Sci.

[14] Nishioka K, Wider C, Vilariño-Güell C, Soto-Ortolaza AI, Lincoln SJ, Kachergus JM (2010). Association of alpha-, beta-, and gamma-Synuclein with diffuse lewy body disease. Arch Neurol.

[15] Livak KJ, Schmittgen TD (2001). Analysis of relative gene expression data using real-time quantitative PCR and the 2(−Delta Delta C(T)) method. Methods.

[16] GTEx Consortium, Laboratory, Data Analysis &Coordinating Center (LDACC)—Analysis Working Group; Statistical Methods groups—Analysis Working Group; Enhancing GTEx (eGTEx) groups; NIH Common Fund; NIH/NCI; NIH/NHGRI (2017). Genetic effects on gene expression across human tissues. Nature.

[17] Lappalainen T, Sammeth M, Friedländer MR, ’t Hoen PA, Monlong J, Rivas MA (2013). Transcriptome and genome sequencing uncovers functional variation in humans. Nature.

[18] Gong J, Mei S, Liu C, Xiang Y, Ye Y, Zhang Z (2018). PancanQTL: systematic identification of cis-eQTLs and trans-eQTLs in 33 cancer types. Nucleic Acids Res.

[19] Goh G, Choi M (2012). Application of whole exome sequencing to identify disease-causing variants in inherited human diseases. Genomics Inform.

[20] Hoepken HH, Gispert S, Azizov M, Klinkenberg M, Ricciardi F, Kurz A (2008). Parkinson patient fibroblasts show increased alpha-synuclein expression. Exp Neurol.

[21] Chen YC, Hsiao CJ, Jung CC, Hu HH, Chen JH, Lee WC (2016). Performance metrics for selecting single nucleotide polymorphisms in late-onset Alzheimer’s disease. Sci Rep.

[22] Hashimoto R, Ikeda M, Ohi K, Yasuda Y, Yamamori H, Fukumoto M (2013). Genome-wide association study of cognitive decline in schizophrenia. Am J Psychiatry.

[23] Hu X, Wetsel RA, Ramos TN (2014). Carboxypeptidase N-deficient mice present with polymorphic disease phenotypes on induction of experimental autoimmune encephalomyelitis. Immunobiology.

[24] Woo J, Kwon SK, Nam J, Choi S, Takahashi H, Krueger D (2013). The adhesion protein IgSF9b is coupled to neuroligin 2 via S-SCAM to promote inhibitory synapse development. J Cell Biol.

[25] Mandolesi G, Gentile A, Musella A (2015). Synaptopathy connects inflammation and neurodegeneration in multiple sclerosis. Nat Rev Neurol.

[26] Eroglu C, Barres BA (2010). Regulation of synaptic connectivity by glia. Nature.

[27] Shaw PJ, Lamkanfi M, Kanneganti TD (2010). NOD-like receptor (NLR) signaling beyond the inflammasome. Eur J Immunol.

[28] Sadovnick AD, Traboulsee AL, Zhao Y, Bernales CQ, Encarnacion M, Ross JP (2017). Genetic modifiers of multiple sclerosis progression, severity and onset. Clin Immunol.

[29] Eilbeck K, Lewis SE, Mungall CJ, Yandell M, Stein L, Durbin R (2005). The sequence ontology: a tool for the unification of genome annotations. Genome Biol.

[30] Cazaly E, Charlesworth J, Dickinson JL, Holloway AF (2015). Genetic determinants of epigenetic patterns: providing insight into disease. Mol Med.

