# Supplementary material for: Exome sequencing study in patients with multiple sclerosis reveals variants associated with disease course
Source: J Neuroinflammation. 2018 Sep 14;15:265. doi: 10.1186/s12974-018-1307-1 (PMC6138928; doi:10.1186/s12974-018-1307-1)
Supplement: Supplementary file 1 — Table S1. Demographic and clinical characteristics of the MS patients with benign and aggressive disease courses. Figure S1. IGSF9B expression levels in PBMC from MS patients stratified according to the genetic variant associated with disease course. Figure S2. GTEx eQTLs of rs10894768 associations with IGSF9B expression in thyroid and pancreas tissues. (DOC 551 kb) [file 12974_2018_1307_MOESM1_ESM.doc]

**Supplementary Material**

**Table S1.** Demographic and clinical characteristics of the MS patients with benign and aggressive disease courses.

| **Characteristics** | **Benign** | **Aggressive** |
| --- | --- | --- |
| *Discovery cohort* |  |  |
| n | 10 | 10 |
| Female/male (% women) | 9/1 (90.0%) | 9/1 (90.0%) |
| Age at onset (years)^a^ | 30.7 (9.4) | 25.9 (6.5) |
| Disease duration (years)^a,b^ | 18.6 (3.2) | 3.9 (0.9) |
| MSSS^a,b^ | 1.2 (0.6) | 9.0 (0.9) |
| RO/PPMS | 10/0 | 10/0 |
| *First validation cohort* |  |  |
| n | 107 | 87 |
| Female/male (% women) | 82/25 (76.6%) | 60/27 (68.9%) |
| Age at onset (years)^a^ | 28.6 (8.9) | 36.0 (10.6) |
| Disease duration (years)^a,b^ | 24.0 (6.8) | 9.1 (5.6) |
| MSSS^a,b^ | 0.9 (1.1) | 8.7 (1.3) |
| RO/PPMS | 107/0 | 80/7 |
| *Second validation cohort* |  |  |
| n | 224 | 33 |
| Female/male (% women) | 178/46 (79.5%) | 24/9 (72.7%) |
| Age at onset (years)^a^ | 26.7 (7.4) | 40.4 (10.7) |
| Disease duration (years)^a,b^ | 23.6 (7.5) | 3.6 (1.1) |
| MSSS^a,b^ | 1.1 (1.3) | 9.5 (0.3) |
| RO/PPMS | 203/21 | 13/20 |

^a^ Data are expressed as mean (standard deviation). ^b^Refers to disease duration and MSSS at the time of the last visit. MSSS: multiple sclerosis severity score. RO: relapse-onset multiple sclerosis, which includes patients with relapsing-remitting and secondary progressive disease. PPMS: primary progressive multiple sclerosis.

**Figure S1.** *IGSF9B* expression levels in PBMC from MS patients stratified according to the genetic variant associated with disease course.

Messenger RNA expression levels for *IGSF9B* were determined by real-time PCR relative quantification in PBMC from a subgroup of untreated MS patients with aggressive disease course carrying the minor allele (MA) for the polymorphism associated with disease course (rs10894768) (n=4) and compared with non-carrier patients (n=3). Results are expressed as fold-change in gene expression in carriers of the minor allele for rs10894768 relative to non-carriers (baseline). All results are expressed as the mean ± SEM.

**Figure S2.** GTEx eQTLs of rs10894768 associations with *IGSF9B* expression in thyroid and pancreas tissues.

**Thyroid**

**Pancreas**

**
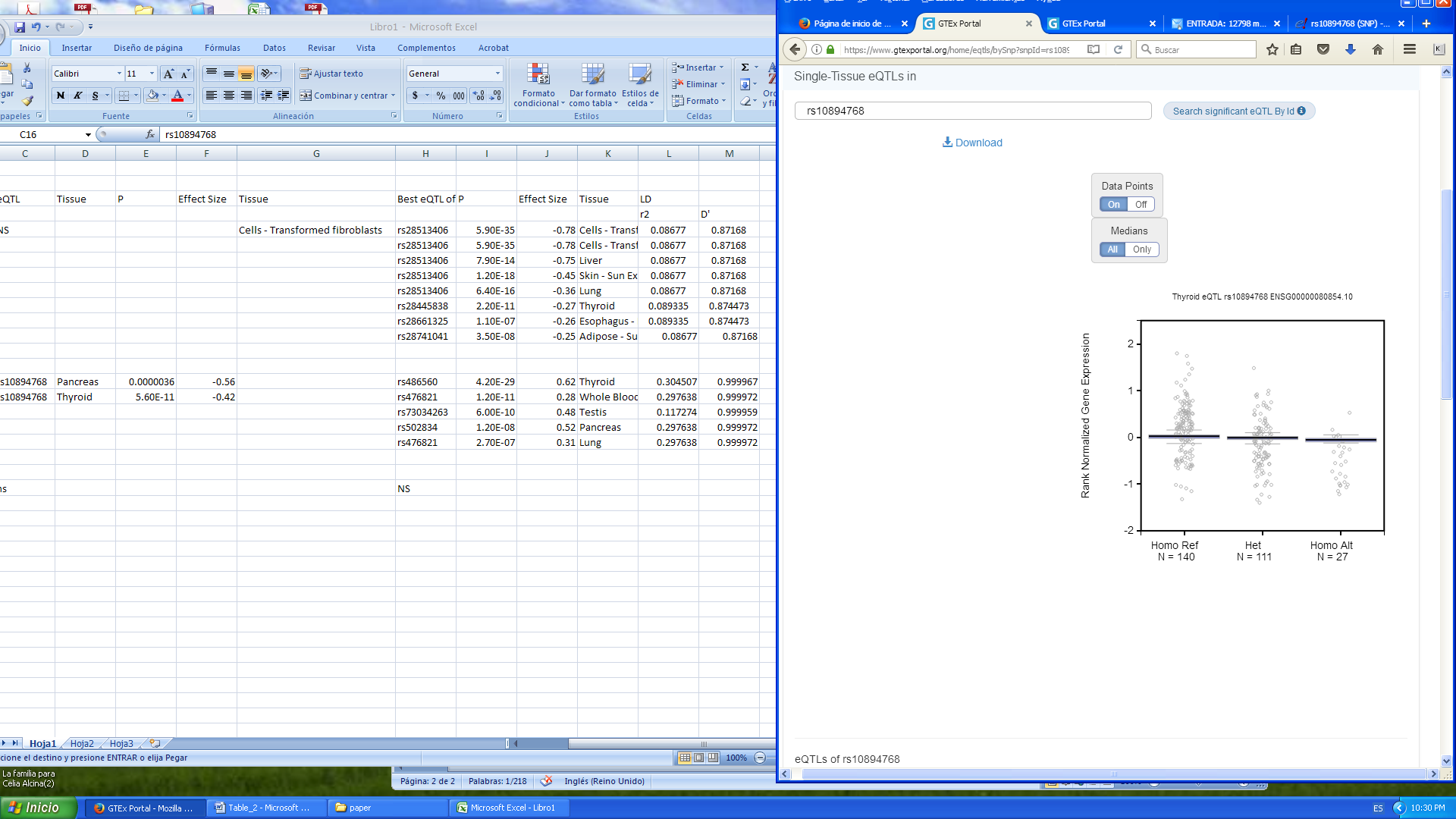

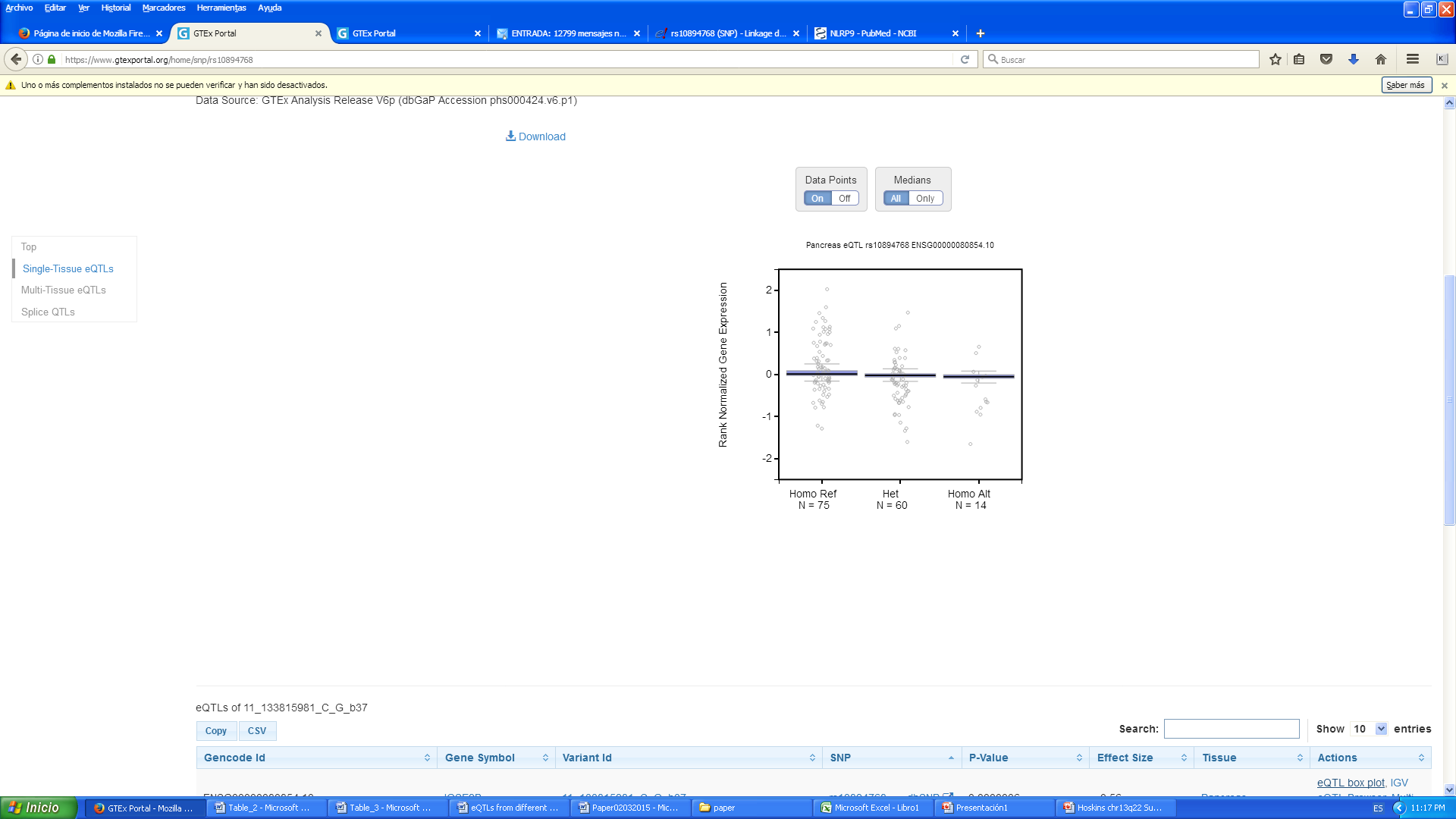
**

Rank normalized of

*IGSF9B* expression

Rank normalized of

*IGSF9B* expression

CC CG GG

n=75 n=60 n=14

CC CG GG

n=140 n=111 n=27

Boxplots representing the expression values of *IGSF9B* in relation with genotypes of the rs10894768 variant associated with disease course. A significant correlation is observed between rs10894768 genotypes and *IGSF9B* expression in pancreatic (p=3.6x10^-6^) and thyroid (p=5.6x10^-11^) tissues.
